# Economic evaluation of the “paramedics and palliative care: bringing vital services to Canadians” program compared to the status quo

**DOI:** 10.1007/s43678-024-00738-9

**Published:** 2024-07-31

**Authors:** J. E. Tarride, D. Stennett, A. C. Coronado, R. Shaw Moxam, J. H. E. Yong, A. J. E. Carter, C. Cameron, F. Xie, M. Grignon, H. Seow, G. Blackhouse, in collaboration with the Paramedics and Palliative Care: Bringing Vital Services to Canadians Paramedic Partners

**Affiliations:** 1 Centre for Health Economics and Policy Analysis (CHEPA), McMaster University, Hamilton, ON Canada; 2 Department of Health Research Methods, Evidence and Impact, Faculty of Health Sciences, McMaster University, Hamilton, ON Canada; 3 Programs for Assessment of Technology in Health (PATH), The Research Institute of St. Joe’s Hamilton, St. Joseph’s Healthcare Hamilton, Hamilton, ON Canada; 4 Healthcare Excellence Canada, Ottawa, ON Canada; 5 Canadian Partnership Against Cancer, Toronto, ON Canada; 6 Department of Emergency Medicine, Dalhousie University, Halifax, NS Canada; 7 Emergency Health Services Nova Scotia, Halifax, NS Canada; 8 Schulich School of Medicine, Western University, London, ON Canada; 9 Canadian Virtual Hospice, Winnipeg, MB Canada; 10 McNally Project for Paramedicine Research, Toronto, ON Canada; 11 Department of Paramedicine, Faculty of Medicine, Nursing and Health Sciences, Monash University, Melbourne, Australia; 12 Department of Health, Aging and Society, Faculty of Social Sciences, McMaster University, Hamilton, ON Canada; 13 Department of Oncology, Faculty of Health Sciences, McMaster University, Hamilton, ON Canada

**Keywords:** Paramedic, Palliative, Economics, Cost-effectiveness, Paramédical, Soins palliatifs, Economie, Rentabilité

## Abstract

**Objective:**

Based on programs implemented in 2011–2013 in three Canadian provinces to improve the support paramedics provide to people receiving palliative care, the Canadian Partnership Against Cancer and Healthcare Excellence Canada provided support and funding from 2018 to 2022 to spread this approach in Canada. The study objectives were to conduct an economic evaluation of “the Program” compared to the status quo.

**Methods:**

A probabilistic decision analytic model was used to compare the expected costs, the quality-adjusted life years (QALYs) and the return on investment associated with the Program compared to the status quo from a publicly funded healthcare payer perspective. Effectiveness data and Program costs, expressed in 2022 Canadian dollars, from each jurisdiction were supplemented with literature data. Probabilistic sensitivity analyses varying key model assumptions were conducted.

**Results:**

Analyses of 5416 9-1-1 calls from five jurisdictions where paramedics provided support to people with palliative care needs between April 1, 2020 and March 31, 2022 indicated that 60% of the 9-1-1 calls under the Program enabled people to avoid transport to the emergency department and receive palliative care at home. Treating people at home saved paramedics an average of 31 min (range from 15 to 67). The Program was associated with cost savings of $2773 (95% confidence interval [CI] $1539–$4352) and an additional 0.00069 QALYs (95% CI 0.00024–0.00137) per 9-1-1 palliative care call. The Program return on investment was $4.6 for every $1 invested. Threshold analyses indicated that in order to be cost saving, 33% of 9-1-1 calls should be treated at home under the Program, the Program should generate a minimum of 97 calls per year with each call costing no more than $2773.

**Conclusion:**

The Program was cost-effective in the majority of the scenarios examined. These results support the implementation of paramedic-based palliative care at home programs in Canada.

**Supplementary Information:**

The online version contains supplementary material available at 10.1007/s43678-024-00738-9.

## Résumé

**Objectifs:**

En fonction des programmes mis en œuvre en 2011-2013 dans trois provinces canadiennes pour améliorer le soutien que les ambulanciers paramédicaux fournissent aux personnes recevant des soins palliatifs. le Partenariat canadien contre le cancer et Excellence des soins de santé Canada a fourni un soutien et du financement de 2018 à 2022 pour diffuser cette approche au Canada. Les objectifs de l’étude étaient d’effectuer une évaluation économique du « programme » par rapport au statu quo.

**Méthodes:**

Un modèle probabiliste d’analyse décisionnelle a été utilisé pour comparer les coûts prévus, les années de vie ajustées en fonction de la qualité (AVAQ) et le rendement du capital investi associés au Programme par rapport au statu quo du point de vue des payeurs de soins de santé financés par l’État. Les données sur l’efficacité et les coûts du Programme, exprimés en dollars canadiens de 2022, de chaque administration ont été complétées par des données documentaires. Des analyses probabilistes de sensibilité ont été effectuées en fonction de diverses hypothèses clés du modèle.

**Résultats:**

Des analyses de 5416 appels 9-1-1 provenant de cinq administrations où des ambulanciers paramédicaux ont fourni du soutien aux personnes ayant des besoins en soins palliatifs entre le 1er avril 2020 et le 31 mars 2022 ont indiqué que 60 % des 9Les appels 1-1 dans le cadre du Programme ont permis aux gens d’éviter le transport vers les urgences et de recevoir des soins palliatifs à domicile. Le traitement à domicile a permis aux ambulanciers paramédicaux d’économiser en moyenne 31 minutes (de 15 à 67 minutes). Le programme a permis de réaliser des économies de 2 773 $ (intervalle de confiance [IC] de 95 %, de 1 539 $ à 4 352 $) et de 0,00069 AVAQ supplémentaires (IC à 95 %, de 0,00024 à 0,00137) par appel de soins palliatifs 9-1-1. Le rendement du capital investi du Programme était de 4,6 $ pour chaque dollar investi. Les analyses des seuils ont indiqué que pour réaliser des économies, 33 % des appels 9-1-1 devraient être traités à domicile dans le cadre du Programme, le Programme devrait générer un minimum de 97 appels par année, chaque appel ne dépassant pas 2773 $.

**Conclusion:**

Le Programme a été rentable dans la majorité des scénarios examinés. Ces résultats appuient la mise en œuvre de programmes de soins palliatifs paramédicaux à domicile au Canada.

## Clinician’s capsule

**Table:** 

***What is known about the topic?***
Paramedic-based palliative care at home increases comfort and quality of life of people receiving palliative care and their families.
***What did this study ask?***
What are the costs and benefits associated with implementing a paramedic-based palliative care at home (the Program) in Canada?
***What did this study find?***
Based on 5416 9-1-1 calls from people requesting palliative support from April 1, 2020, to March 31, 2022, the Program was associated with cost savings of $2,280 per 9-1-1 call.
***Why does this study matter to clinicians?***
With increased pressure on Canadian emergency departments, these economic results support the implementation of paramedic-based palliative care at home programs.

## Introduction

The majority of those 334,000 individuals who die annually in Canada [1] are considered to have a palliative diagnosis or are close to the end of life. In the last two weeks of their life, 40% of people with palliative goals of care visit the emergency department [2] and paramedics facilitate more than half of these visits [3]. Recognizing that transport is often not optimal [4], paramedics have expressed a desire to support these people differently, which has led to the development and implementation of innovative palliative programs in 2011–2013 in Nova Scotia, Alberta, and Prince Edward Island [5]. Under these programs, following a 9-1-1 call, paramedics were empowered to support people at home without a transport to the emergency department, if appropriate.

The inclusion of the palliative approach is now part of standard of care for all paramedics responding within the 9-1-1 system and this response does not represent new/separate staffing. The main changes implemented to support this change in practice included (1) making goals of care accessible and known to paramedics, (2) providing education in palliative care approaches, (3) expanding paramedics’ tools, skills, and resources to provide a palliative approach to care (e.g., new clinical practice guidelines and additional medications in some jurisdictions), and (4) empowering non-transport through formalized pathways and protocols and increased communication between paramedics and other members of the community care team (home care, palliative consult service, etc.). Compared to the status quo, in which people were transported to an emergency department unless the person receiving palliative care or family refuses, these programs have several benefits including improving access to palliative care, increasing comfort and quality of life of people receiving palliative care and their families, improving paramedic and palliative care provider experience and fit with professional identity, reducing emergency department visits and optimizing paramedics’ time utilization [6–8].

Based on this experience, and in collaboration with several healthcare delivery providers (the “Paramedic Partners”), the Canadian Partnership Against Cancer (the Partnership) and Healthcare Excellence Canada (HEC) provided support and funding between 2018 and 2022 under the Paramedics and Palliative Care: Bringing Vital Services to Canadians program (“the Program”) to spread this approach in Canada as part of a quality improvement approach [5, 9]. While the key elements noted above were maintained across Paramedic Partners, jurisdictional flexibility led to locally tailored implementation as detailed in Appendix 1. To provide evidence for the broader implementation in Canada of paramedic-based models of palliative care in the home, the study objectives were to conduct an economic evaluation of the Program compared to the status quo.

## Methods

### Study design

Similar to other economic evaluations of quality improvement initiatives [10–15], decision analytic techniques [16–18] combining multiple sources of information were used to compare the Program and the status quo in terms of costs and benefits. The study conduct and reporting followed Canadian [19] and international guidelines [16, 17, 20] for economic evaluations of healthcare programs. The detailed methods are presented in an online document and briefly summarized below.

### Study population

The study population consisted of people with palliative goals of care who called 9-1-1 for at-home paramedic support in designated areas served by British Columbia Emergency Health Services, Saskatchewan Health Authority Regina Area, Interlake-Eastern Regional Health Authority in Manitoba, Extra Mural Program & Ambulance New Brunswick, and Eastern Health in Newfoundland and Labrador (the “Paramedic Partners”) [9].

### Model structure

**Fig. 1:**
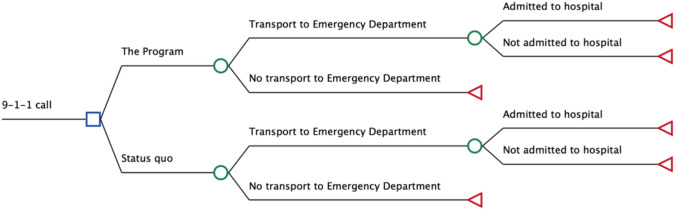
Model structure

As shown in Fig. 1, under the status quo scenario, people calling 9-1-1 for palliative care were automatically transported to the emergency department for medical management unless transportation is refused by them or family. Under the Program, when possible and desired by the person requesting care and family, those people received palliative care in their home by trained paramedics; a proportion desired transport to the emergency department or required it for adequate management. People transported to the emergency department were discharged home or hospitalized. The time horizon of the model was two weeks which corresponded to the average length of stay of a palliative care hospitalization in Canada in fiscal year 2021/2022 (i.e., 15.5 days) [21].

### Data sources and variables

Aggregated data collected by each Paramedic Partner from April 1, 2020, to March 31, 2022, were used to inform the percentage of 9-1-1 calls resulting in people being treated at home under the Program, the time that paramedics spent answering the call and the top 5 complaints leading to a call. The study authors surveyed the Paramedic Partners to estimate the Program set-up, implementation, and ambulance trip costs from October 31, 2018 to March 31, 2021 (the latest available data at the time of the survey).

Historical data from New Brunswick [6] were used to model the proportion of 9-1-1 calls from people receiving palliative care resulting in an emergency department transport (90%) in the absence of the Program. Following an emergency department visit under the status quo scenario, it was assumed that 66% of people would be hospitalized based on a 2023 Canadian report on 89,000 people receiving palliative care in fiscal year 2021/2022 [21]. Since some people will be treated at home and not transported to the hospital under the Program, a higher rate of hospital admissions following an emergency department visit was assumed for the Program (i.e., 83% as the mid-point between the status quo of 66% [21] and the maximum percent of emergency department transports that can result in a hospitalization, 100%).

Published data were used to model the impact of the Program on quality of life. The baseline quality of life of a person with palliative care needs was assumed to be 0.37 [22] anchored on 0 representing death and 1 perfect health. Decrease in quality of life due to a visit to the emergency department (decrease of 0.018 [23]) or hospitalization (decrease of 0.06 per day of hospitalization [24]) was applied to the baseline value of 0.37. These values were combined with the model duration of two weeks to calculate the quality-adjusted life years (QALYs) associated with each intervention. Details on the QALY calculations can be found in the online document. The costs in 2022 Canadian dollars associated with a visit to the emergency department ($333) [25] or a palliative care hospitalization ($9709) [26] were taken from the Canadian Institute for Health Information.

### Economic analysis

Cost–benefit and cost–utility analyses were conducted to evaluate the Program. In a cost–benefit analysis [27], outcomes (e.g., reduction in emergency department transport) are expressed in terms of monetary benefits (e.g., savings in emergency department visits) which allows the calculation of the return on investment (difference between the healthcare cost savings associated with the Program and the Program cost divided by the Program cost). In cost–utility analyses [27], the results are expressed in terms of incremental cost per QALY gained. Several sensitivity analyses were conducted to assess the impact of changing key model inputs on the model results (see online document). All analyses were probabilistic and undertaken from a publicly funded healthcare payer perspective.

### Ethics approval

For this quality improvement initiative, we received an exemption from the Ottawa Health Science Network Research Ethics Board.

## Results

### Proportion of people receiving palliative care treated at home and ambulance time

Overall, the Paramedic Partners who implemented the Program responded to 5416 9-1-1 calls for people with palliative goals of care from April 1, 2020 to March 31, 2022. Top reasons for the calls were breathing problems/dyspnea, pain, sick person, unconscious/fainting, and falls. Sixty percent of the calls enabled people receiving palliative care to remain in their homes without transportation to the emergency department. Treating people at home saved paramedics an average of 31 min (range from 15 to 67). Reduced emergency department transports and time on task were observed in both urban and rural areas, with some variation between Paramedic Partners. Table 1 presents the combined data and ranges from the five Paramedic Partners.

**Table 1:** Proportion of people receiving palliative care treated at home under the Program and time committed to the 9-1-1 call (April 1, 2020, to March 31, 2022): means and range observed among the five paramedic partners

Variables	Overall	Urban setting^a^	Rural setting^a^
Cumulative number of 9-1-1 palliative care calls [minimum–maximum across paramedic programs]	5,416 [117–2917]	4044 [24–2472]	1343 [32–445]
Proportion of 9-1-1 palliative calls resulting in people being treated at home instead of being transported to the emergency department: Mean [minimum–maximum across paramedic programs]	60% [30–80%]	63% [32%–78%]	54%[29–89%]
Time committed to 9-1-1 palliative care call in minutes: means [minimum–maximum across paramedic programs]			
Calls that resulted in transport to the emergency department	93 [89–119]	100 [85–120]	91 [61–98]
Calls that did not result in transport to the emergency department	61 [49–76]	56 [42–78]	57 [42–61]
Difference in minutes between calls that did and did not result to a transport to the emergency department	31 [15–67]	44 [11–68]	34 [17–42]

Source: Paramedic Partner Programs who reported aggregated indicators on an annual basis to the Partnership, which in turn sent the aggregated data to the study authors from McMaster University

^a^There were 29 calls that were not identified as taking place in either an urban or rural setting and were excluded from the urban/rural analyses. The classification urban/rural was provided by the Paramedic Partner and definitions of urban/rural were dependent on the jurisdictions

### Program cost per 9-1-1 palliative care call

The average cost to implement the Program was $266,855 per year. The Program generated, on average, 542 9-1-1 calls per year, per Paramedic Partner, with a call costing $493 (i.e., $266,855/542 calls per year). Due to variations in Paramedic Partner costs and call volumes, the cost per call ranged from $270 to $3869 as shown in Table 2.

**Table 2:** Program cost per Paramedic Partner per year, number of 9-1-1 calls for people with palliative care goals and, Program cost per 9-1-1 palliative care call (2022 Canadian dollars): means and range observed among the five Paramedic Partners

	Per paramedic partner per year
Program cost (A) [minimum–maximum across paramedic programs]*	$266,855 [$118,625–$355,720]
Compensation	$154,307 [$77,581–$260,366]
Meetings and travel	$6185 [$5632–$10,851]
Professional services	$9796 [$0–$35,862]
Education	$70,612 [$6248–$238,039]
Equipment	$19,599 [$0–$59,382]
Supplies	$4895 [$779–$6055]
Other	$1461 [$0–$6633]
Number of 9-1-1 palliative care calls (B) [minimum–maximum across paramedic programs]*	542 [59–1459]
Program cost per 9-1-1 palliative care call (A/B) [minimum–maximum across paramedic programs]**	$492.72 [$270–$3869]

Source: Paramedic Partner Programs

*Based on 2.5 years of costing data from the five Paramedic Partners (each Partner contributed data from October 2018 to March 31, 2021)

**Based on 2 years of 9-1-1 calls for people with palliative care goals from April 1, 2020, to March 31, 2022 (each Paramedic Partner contributed to the data)

### Economic results

Compared to the status quo, the Program was estimated to reduce the health care costs associated with emergency department visits and hospitalizations by $2773 per 9-1-1 palliative care call (95% CI $1539–$4352). This offset the Program costs of $493 per call. The overall cost savings associated with the Program were calculated at $2280 (95% confidence interval [CI] $1030–$3871) or approximately $12.3 million for the 5416 calls received under the Program. The return on investment was $4.6 for every dollar invested (i.e., $2280/$493). The Program also produced 0.00069 (95% CI 0.00024–0.00137) more QALYs per 9–1-1 call (i.e., the equivalent of 6 h of perfect health) compared to the status quo. Table 3 presents the details.

**Table 3:** Probabilistic results: base case analysis (per 9-1-1 call for people with palliative care goals; 2022 Canadian dollars)

	Program (means and 95% confidence intervals)	Status Quo (means and 95% confidence intervals)	Difference (means and 95% confidence intervals)
Program costs (A)	$493 ($320–$704)	$0.00 ($0–$0)	$493 ($320–$70 4)
Total health care cost (B)	$3538 ($2372–$4911)	$6311 ($4117–$9052)	− $2773 (− $4352–$1539)
Ambulance calls*	$216 ($140–$308)	$262 ($171–$384)	− $46 (− $69 to − $28)
Emergency department visits	$132 ($87–$192)	$300 ($196–$437)	− $168 (− $248 to − $108)
Hospitalizations	$3190 ($2024–$4656)	$5749 ($3566–$8480)	− $2559 (− $4130 to − $1330)
Overall costs (A + B)	$4031 ($2851–$5500)	$6311 ($4117–$9052)	− $2280** (− $3871 to − $1030)
Expected QALYs**	0.01487 (0.01102–0.01889)	0.01418 (0.01009–0.01830)	0.00069 (0.00024–0.00137)
Return on investment			4.6 ($2280/$493)
Incremental cost-effectiveness ratio (ICER)			Program dominates (less costly and more effective)

Notation: QALY: quality-adjusted life years

*Two of the five Paramedic Partners provided data on ambulance costs ($161 per hour and $186 per hour) for an average of $174 per ambulance hour

**As a reference, over the model time horizon of two weeks (i.e., 15.5 days), the maximum number of QALYs for an individual with a baseline utility of 0.37 is 0.016. An improvement of 0.0069 QALYs correspond to 6 h of perfect life over the model time horizon

Sensitivity analyses results presented in Table 4 shows that the Program was not cost-effective when 30% of people were treated at home or when the Program cost was $3869 per 9-1-1 call, as observed in two jurisdictions. In all other sensitivity analyses, the Program resulted in cost savings ranging from $563 to $3946 per 9-1-1 call. Based on the scenarios simulated, to generate cost savings, at least 33% of 9-1-1 calls should be treated at home instead of transported, the Program should generate a minimum of 97 calls per year and each call should cost no more than $2773. The Program was not cost saving if these criteria were not met.

**Table 4:** Probabilistic sensitivity analyses comparing the Program versus status quo (per 9–1-1 call for people with palliative care goals; 2022 Canadian dollars)

	Incremental costs (includes intervention cost)	Incremental quality-adjusted life years (QALYs)	Probability Program is cost-effective if willingness to pay for a QALY gained is $50,000
Base case analyses	− $2280	0.00069	1.00
Scenario analyses			
Urban-based transportation and ambulance time data	− $2299	0.00069	1.00
Rural-based transportation and ambulance time data	− $2287	0.00069	1.00
Sensitivity analysis around proportion of people treated at home under the Program rather than being transported to the emergency department (60% base case)			
Assume 30% of people treated at home (lowest value observed among the Paramedic Partners)	$283	0.00004	0.30
Assume 40% of people treated at home	− $563	0.00025	0.85
Assume 50% of people treated at home	− $1409	0.00047	1.00
Assume 70% of people treated at home (highest value observed among the paramedic partners)	− $3946	0.00111	1.00
Sensitivity analysis around the proportion of individuals being hospitalized after a transport to the emergency department for the Program (83% base case)			
Assume emergency department to hospital rate for Program is 66%	− $2934	0.00086	1.00
Assume emergency department to hospital rate for Program is 75%	− $2588	0.00077	1.00
Assume emergency department to hospital rate for Program is 90%	− $2011	0.00062	1.00
Assume emergency department to hospital rate for Program is 100%	− $1626	0.00051	1.00
Sensitivity analysis around the intervention cost per call ($493 per call in base case)			
Intervention cost per call is $270 per call (lowest cost per 9-1-1 observed among the Paramedic Partners)	− $2503	0.00069	1.00
Intervention cost per call 20% higher than in base case analysis	− $2182	0.00069	1.00
Intervention cost per call 20% lower than in base case analysis	− $2362	0.00069	1.00
Intervention cost per call is $3869 per call (highest cost per 9-1-1 call observed among the Paramedic Partners)	$1096	0.00069	0.12
Amortizing training costs over three years	− $2440	0.00069	1.00
Include 2019/2020 data for transport rate and ambulance time	− $2377	0.00071	1.00
Assume 87% transport to the emergency department for the control group (status quo)	− $2077	0.00064	1.00
Assume 95% transport to the emergency department for the control group (status quo)	− $2619	0.00077	1.00
Sensitivity analysis around the costs of hospitalization and emergency department visits			
Costs for hospitalization and emergency department visits are 20% higher than in base case analysis	− $2835	0.00069	1.00
Costs for hospitalization and emergency department visits are 20% lower than in base case analysis	− $1818	0.00069	1.00

Notations: QALY: quality-adjusted life years

## Discussion

### Interpretation of findings

This model-based evaluation on more than 5400 9-1-1 calls indicates that the Program overall saves costs. The Program also slightly improves QALYs compared to the status quo although an improvement of 6 h of perfect health over the two-week model duration may be relevant and meaningful for individuals with palliative care needs. The results were driven by a decrease in the number of transports to the emergency department and time responding to a call associated with the Program. However, there were variations in emergency department transport, number of 9-1-1 calls and Program cost observed across Paramedic Partners which highlight the uniqueness in context, geography, and delivery. Therefore, to ensure sustainability, organizations interested in this model of care may wish to establish targets around volume of 9-1-1 calls and emergency department transportation rates that are considerate of their own contexts and monitor them regularly during program implementation and beyond.

### Comparison to previous studies

It is difficult to compare our results with previous studies as there is a lack of economic evaluations of paramedic-led palliative care programs as shown by a 2023 review [28] of 56 studies of emergency medical services and palliative care. Previous evaluations of paramedic-led palliative care programs at home conducted in Canada [7, 8] and elsewhere [28, 29] have traditionally focused on paramedics’ comfort and satisfaction on delivering palliative care and patient and family quality of life, underscoring the need for further studies to evaluate the effectiveness and cost-effectiveness of paramedic-led models of care [28]. Our results are however consistent with the findings of several community-based paramedic interventions which have shown to reduce emergency department visits or hospitalizations in Canada [30–32] and elsewhere [30, 33–40] according to a 2022 review [41] of community paramedic programs. Among the 12 studies evaluating economic outcomes included in this review of 98 studies [41], five studies conducted in Canada showed that community-based paramedic programs were cost saving [31, 42, 43] or cost-effective [44, 45], which is also consistent with our economic results. Positive economic outcomes associated with community-based paramedic models were also observed in the US, Europe and Australia [41].

### Strengths and limitations

Key strengths of this pan-Canadian economic evaluation are using outcome data from more than 5400 9-1-1 palliative care calls and a thorough collection of Program costs from Paramedic Partners. The study limitations reflect the realities and common issues of healthcare delivery research and quality improvement initiatives. For example, the disconnect between paramedic databases and hospital administrative databases prevented the collection of individual-level demographics or the presenting complaint by emergency department. Therefore, hospital re-admissions following transport to the emergency department, subsequent 9-1-1 calls, or time/location of death for people initially treated at home were not available. It was also not possible to determine treatments provided by paramedics or how often each person called. Historical data was relied on in the absence of access to a control group. Furthermore, to derive the cost to implement the Program, we used effectiveness data from April 1, 2020 to March 31, 2022, while the cost data was from October 2018 to March 31, 2021, which may have introduced some bias in our calculations. It was also not possible to conduct a comparison across the Paramedics Partners and the cost-effectiveness of individual Programs remains unknown. To address some of these limitations, we conducted several sensitivity analyses which indicated that the Program was cost-effective in the majority of the scenarios examined. Our model did not include the high patient and family satisfaction associated with the Program [46]. As such, our evaluation, which focused on the Program initial implementation, is likely to overestimate the long-term costs of running the Program as the Program costs will decrease over time. This could underestimate the Program long-term benefits.

### Health systems implications

Under the Program, paramedics played a key role in collaborating with local healthcare providers and by supporting people needing palliative care in urban and rural health systems, especially those in areas with limited palliative home care resources. The Program overall was found to be cost-effective, facilitated efficiencies to the health system through the reduction of emergency department transportations and related hospitalizations, and supported patient-centered palliative care when and where people wanted it. The Paramedic Partners successfully adapted the Program to varying health system contexts and further spread of the Program may show similar benefits, depending on the local context.

### Implications for future research

Efforts should be made to document healthcare resource utilization and place of death following a 9-1-1 palliative care call that did or did not result in an emergency department transport (e.g., by linking paramedic databases with hospital administrative databases). Second, continued monitoring of individual Programs as they mature is also very important as a large component of the Program costs included in our evaluation was related to the cost of training paramedics to provide palliative care at home. Once trained, costs of ongoing staff training and continuing education will likely be lower. It is also anticipated that the proportion of transports to the emergency department would decrease over time as the individual programs mature and become fully operationalized. Future research should evaluate the cost-effectiveness of the Program in other jurisdictions once mature data become available, including the two Paramedic Partners based in Ontario (Ottawa Hospital Research Institute and York Region Paramedic Services) whose data collection was delayed due to the COVID-19 pandemic.

## Conclusions

In a context of crowded emergency departments and considering patient preferences to receive palliative care and die at home [47]], the results of this economic evaluation support the continued implementation and spread of paramedic-based palliative care at home in Canada as the program was found to be cost-effective across the majority of scenarios simulated.

## Supplementary Information

Below is the link to the electronic supplementary material.

Supplementary file1 (DOCX 16 KB)

Supplementary file2 (DOCX 255 KB)
