# Supplementary material for: Economic evaluation of the “paramedics and palliative care: bringing vital services to Canadians” program compared to the status quo
Source: CJEM. 2024 Jul 31;26(9):671–80. doi: 10.1007/s43678-024-00738-9 (PMC11377656; doi:10.1007/s43678-024-00738-9)
Supplement: Supplementary file 1 — Supplementary file1 (DOCX 16 KB) [file 43678_2024_738_MOESM1_ESM.docx]

Appendix 1: Summary of Program Model Components by Paramedic Partner

| **Program Feature** | **NL** | **NB** | **MB** | **SK** | **BC** |
| --- | --- | --- | --- | --- | --- |
| Treatment in place for individuals with  palliative care needs, 24/7/365 | **🗸** | **🗸** | **🗸** | **🗸** | **🗸** |
| Program uses existing paramedic teams (911 response) = palliative care is integrated into existing practices with existing resources | **🗸** (+specialist paramedic) | **🗸** | **🗸** | **🗸** | **🗸** |
| Non-lights and sirens response facilitated | **🗸** | In progress | - | **🗸** | **🗸** |
| Care aligned with the individual’s wishes/goals of care | **🗸** | **🗸** | **🗸** | **🗸** | **🗸** |
| Palliative care delivered by all levels of  paramedic practice (excluding EMR) | **🗸** | **🗸** | **🗸** | **🗸** | **🗸** |
| No user fee if care provided in-home | **🗸** | **🗸** | - | **🗸** | - |
| Access across province, regardless of how rural or remote | Service region | **🗸** | Service region | Service region | **🗸** |
| Program/response may be activated  by healthcare providers | **🗸** | **🗸** | **🗸** | **🗸** | **🗸** |
| Available to pediatrics with palliative care needs | - | **🗸** | **🗸** | **🗸** | **🗸** |
| Goals of care determined prior to the event | **🗸** | **🗸** | **🗸** | - | - |
| Pre-registration is required/mandatory | **🗸** | - | **🗸** | - | - |
| Ability to be pre-registered/utilizes registry (i.e.: Special Patient Program with paramedic services operator, registry maintained with palliative home care) | **🗸** | **🗸** | **🗸** | - | - |
| Additional medications or expanded uses for symptom management in palliative care for PCPs and/or ACPs | **🗸**  ACPs | **🗸**  ACPs & PCPs | **🗸**  ACPs | **🗸**  ACPs | **🗸**  ACPs & PCPs |
| Training in palliative care provided to paramedics | **🗸** | **🗸** | **🗸** | **🗸** | Ongoing |

Notations: NL: Newfoundland and Labrador; NB: New Brunswick; MB: Manitoba; SK: Saskatchewan, BC: British Columbia

Reproduced with permission from Healthcare Excellence Canada and the Canadian Partnership Against Cancer
