# Supplementary material for: Economic evaluation of the “paramedics and palliative care: bringing vital services to Canadians” program compared to the status quo
Source: CJEM. 2024 Jul 31;26(9):671–80. doi: 10.1007/s43678-024-00738-9 (PMC11377656; doi:10.1007/s43678-024-00738-9)
Supplement: Supplementary file 2 — Supplementary file2 (DOCX 255 KB) [file 43678_2024_738_MOESM2_ESM.docx]

**Online document: Economic methods – details.**

The methods are reported following the format of the Canadian guidelines for the economic evaluation of healthcare interventions(1).

1. Type of economic evaluation

A cost-benefit and cost-utility analyses were conducted to evaluate the Program(2). In a cost-benefit analysis, outcomes (e.g., reduction in emergency department transport) are expressed in terms of monetary benefits (e.g. savings in emergency department visits) which allows the calculation of the return on investment (ROI) associated with the Program (difference between the healthcare cost savings associated with the Program and the Program cost divided by the Program cost). In cost-utility analyses, the outcomes are expressed in terms of quality-adjusted-life years (QALYs), where QALYs combine quantify and quality of life (expressed on a 0-1 scale where 0 corresponds to death and 1 to perfect health). Over one year, the maximum number of QALYs that can be generated is 1 (1 year of life with a perfect health).

1. Target population

The study population consists of people with palliative goals of care who called 9-1-1 for paramedic support in designated areas served by British Columbia Emergency Health Services, Saskatchewan Health Authority Regina Area, Interlake-Eastern Regional Health Authority in Manitoba, Extra Mural Program & Ambulance New Brunswick, and Eastern Health in Newfoundland and Labrador(3) (the Paramedic Partners). The individual requesting care (not paramedics) determined if they wanted to take a palliative approach. This could have been previously documented and kept in the home/on file or the individual and family/caregivers would decide this approach once the paramedics arrived.

1. Comparators

The comparator was the status quo in the absence of the Program (e.g. following a 9-1-1 call), people receiving palliative care are automatically transported to an emergency department unless the individual/family refuses.

1. Perspective

This economic evaluation was conducted from a publicly funded healthcare payer Perspective.

1. Time horizon

The time horizon of the model was 15.5 days which corresponds to the average duration of a palliative care hospitalization. This was derived from a report on palliative access in Canada(4) which the average length of stay for hospitalizations was provided for two different categories of hospitalizations. These categories were hospitalizations “with palliative care” (average length of stay of 17 days; 103,033 hospitalizations) and hospitalizations “for palliative care” (average length of stay of 12 days; 44,034 hospitalizations). A weighted average of the length of stays from these categories were used to the derive the length of stay of all palliative care hospitalizations.

1. Discount rate for costs and effects

Because the time horizon was less than a year, discounting of costs and QALYs was not applicable.

1. Modeling

The Figure below presents the structure of the decision tree used to model the costs and consequences associated with the Program and the status quo. Under the status quo scenario, people calling 9-1-1 for palliative care are automatically transported to the emergency department for medical management unless transportation to the emergency department is refused by the individual or family. Under the Program, when possible and desired by the person requesting care and family, those people receive palliative care in their home by trained paramedics; a proportion will desire transport to the emergency department or will require it for adequate management. People transported to the emergency department can be discharged home or hospitalized.

1. Effectiveness

Based on the Paramedic Partner data and public data, the model considered three effectiveness measures: 1) proportion of emergency department transports; 2) time spent by paramedics on call; 3) proportion of emergency department transports that resulted in an hospital admission.

- 1. Proportion of people treated at home under the Program

For the Program, the proportion of people treated at home under the Program instead of being transported o the emergency department and, time on task for palliative care calls that did or did not result in a transport to the emergency department were collected by the Paramedic Partners and reported using a standardized form. While the proportion of people treated at home and time on task data were also available for fiscal year (FY) 2019/2020 for four of the five Paramedic Partners, the base case analyses exclude FY 2019/2020 as some programs started in the middle of the year and due to potential differences in transport rates before and after the start of the COVID-19 pandemic in March/April 2020. The overall proportion of people treated at home was calculated by first estimating proportion of people treated at home for each of the five individual jurisdiction over the study period (April 1, 2020, to March 31, 2022). A weighted average of the proportion of people treated at home was then estimated by weighing each of the five individual jurisdiction proportions of people treated at home by the total number of 9-1-1 palliative calls over the study period.

Based on historical data from New Brunswick prior to the implementation of the Program in this province, the base case analyses assumes for the status quo that 10% of people will be treated at home while 90% of 9-1-1 palliative care calls would result in transportation to the emergency department.(5) Three Paramedic Partners confirmed that this proportion was representative of their jurisdictions and sensitivity analyses were conducted by using the lower (87%) and highest (95%) emergency department transportation rate reported by the other two Paramedic Partners.

Paramedic time on task was estimated separately for calls where people were transferred to emergency department and for calls where transport to emergency department did not occur. Data on time on call was collected for each jurisdiction annually. The overall time on task for calls with transport was estimated by weighing the average time on task for calls with transport to emergency department for each jurisdiction by the number of 9-1-1 palliative care calls with hospital transport for each jurisdiction. The overall time on task for 9-1-1 palliative calls that did not result in transport to emergency department was estimated in a similar manner. Paramedic Partner data on time on task when the 9-1-1 palliative care call resulted in transport to the emergency department were applied to the status quo.

- 1. Proportion of people hospitalized after being transported to the emergency department

The proportion of emergency department transports that resulted in an admission to hospital (66%) under the status quo was based on the 2023 report from the Canadian Institute for Health Information (CIHI) on access to palliative care in Canada.(4)

While the Program aims to predominantly only transport people with palliative care needs who paramedics determine require hospitalization to the emergency department (i.e., 100% of transported people are hospitalized), there are circumstances in which transported people may not be admitted to the hospital. For example, this may happen when care cannot be provided by the paramedics (e.g., narcotic prescriptions), when the person requests transportation or when support at home is unavailable or limited(6) (e.g., family feels they need more support from the emergency department). To acknowledge this and in the absence of linked paramedic data to hospital databases, the base case analyses assume that the Program would see 83% of emergency department visits result in hospitalization (the mid-point between status quo of 66%(4) and the maximum percentage of transports that could result in hospitalization, 100%).

1. Measurement and valuation of outcomes

For the cost-benefit analysis, the outcomes were expressed in terms of monetarized benefits related to a decrease in emergency department transports or hospitalization. In the cost-utility analysis, the outcomes are expressed in terms of quality-adjusted life-years (QALYs) over the time horizon of the model. QALYs combine quantity of life with quality of life where quality of life is represented by a utility score (anchoring on 0 representing death and 1 perfect health). Over one year, the maximum number of QALYs is 1 (1 year of life with a perfect health utility of 1). For the QALY calculations, a baseline utility of 0.37 was used for people receiving palliative care(7). Decreases in utility of 0.018 for an emergency department visit(8) and 0.06 per day of hospitalization(9) were applied for the duration of each emergency department visit (14.5 hours(10)) and palliative care hospitalization (15.5 days) based on CIHI data. QALYs were derived by combining these utilities over the model time horizon. For example, over the time horizon of the model of 15.5 days, the maximum number of QALYs for people receiving palliative care with a baseline utility of .37 was 0.016 (utility of 0.37*15.5 days/365 days). The QALYs for someone who would be transported to the emergency department were calculated at 0.016 (i.e., baseline QALY of 0.016 – 0.00003 QALY due to a decrease in quality of life related to the admission to the emergency department for 14.5 hours or 0.6 days; i.e. 0.018*0.60 days/365 days). Someone transported to the emergency department and admitted to the hospital would have a QALY of 0.013 (0.016 baseline QALYs – 0.00003 QALYs due to the admission to the emergency department – 0.0025 QALYs due to decrease in quality of life related to a 15.5-day hospitalization; i.e. 0.06*15.5 days/365 days).

1. Resource use and costs

Costs included the expenses to implement the Program (intervention costs), the costs associated with sending an ambulance to answer a 9-1-1 call (ambulance costs) and the costs associated with an emergency department visit or an hospital admission.

- 1. Program and ambulance costs

To estimate the intervention and ambulance costs, a survey was developed (Appendix 2) and sent to each Paramedic Partner in November 2021. The survey collected information from October 2018 (start of the program in some jurisdictions) to March 31, 2021 (latest data available at time of survey) on the costs related to training-related meetings/travel, professional services, education, equipment, supplies and whether financial compensation was provided to the paramedics if their training occurred outside of work time, or whether the Paramedic Partners needed to backfill paramedics if training was done during work hours. One Paramedic Partner completed the survey in summer of 2022 and therefore was able to provide costing information for fiscal 2021/2022 as well. The last section of the survey asked questions on the cost of ambulance services (purchasing price of ambulance amortized over 5 years, personal and operating costs) and the number of ambulance service hours, which were used to derive a unit cost per minute of ambulance use. The ambulance cost per minute was multiplied by the time committed to the call to derive the ambulance cost per 9-1-1 palliative care call.

The survey data were reviewed with each Paramedic Partner and a cost per 9-1-1 palliative care call was determined by dividing across all Paramedic Partners the average Program costs per year by the average number of 9-1-1 palliative care calls per year. 9-1-1

- 1. Emergency department and hospitalization costs

Data from the Canadian Institute for Health Information was used for the costs associated with an emergency department visit ($333)(11) and a palliative care hospitalization ($9,709)(12) which were expressed in 2022 dollars using the consumer price index of Statistics Canada.(13)

1. Analysis

As recommended by the Canadian guidelines for the economic evaluations of healthcare technologies,(1) base case and sensitivity analyses were probabilistic, in which the model results were simulated 5,000 times with values from model input variables being drawn from distributions specific to each model parameter(14). Probability distributions were parameterized using empirical data according to good practices.(14) Cost parameters were parameterized using gamma distributions. Gamma distributions are defined by alpha and beta parameters, which are derived from the parameters mean and standard errors. In absence of dispersion data for the cost per day in hospitalization and emergency visit, the standard error of the mean was assumed to be 20% of the mean value (this means that the alpha parameters are always 25 independently of the mean value as can been see in section 11.1).

Probability variables and utility variables were parameterized using beta distributions. Beta distributions have two parameters alpha and beta and can be thought as events (i.e., alpha) and non-events (i.e., beta). For example, for emergency department transportation rate, the total number of 9-1-1 calls that led to transport and the total number of calls that did not lead leading to emergency department transport observed in the study period were used to define the alpha (2,155) and beta (3,266) parameters. For other binary variables such as the probability of admission to hospital after an emergency department transport, a population of 100 was assumed when defining alpha (e.g. number of hospital admission following emergency department transport) and beta (100 – alpha) parameters.

The results of the 5,000 Monte Carlo simulations were used to summarize the uncertainty associated with the results. Confidence intervals around the base case results were estimated by taking the 2.5% and 97.5% percentile of outcomes (costs, QALYS) of the 5,000 Monte Carlo simulations. For the cost-benefit analysis, the proportion of simulations where the net benefit (i.e., healthcare savings due to Program minus Program costs) was positive was determined. For the cost-utility analysis, the probability that the Program was cost-effective was determined as the proportion of simulations under a $50,000/QALY gained willingness to pay threshold used by the Canadian Agency for Drugs and Technologies in Health.(15)

- 1. Base case analyses

The table below summarizes the model input parameters and their associated distributions which were used in the probabilistic analysis for the base case analyses.

| Variable | Model value | Distribution |
| --- | --- | --- |
| Program: Emergency department transportation Rate | 0.40 | Beta (alpha = 2,155; beta = 3,266) |
| Status quo: 3 Transportation rate | 0.90 | Beta (alpha = 90, beta = 10) |
| Program: proportion of emergency department visits that result in hospitalization | 0.83 | Beta (alpha = 83, beta = 17) |
| Status quo: Proportion of emergency department visits that result in hospitalization | 0.66 | Beta (alpha = 66, beta = 34) |
| Time committed to calls that result in transport to emergency department (minutes) | 98.5 | Normal (mean = 98.5; SE = 2.4) |
| Time committed to calls that do not result in transport to emergency department (minutes) | 58.8 | Normal (mean = 58.9; SE = 1.8) |
| Program cost per call | $493 | Gamma (alpha = 25; beta = 20) |
| Cost per emergency department visit | $333 | Gamma (alpha = 25; beta = 13) |
| Cost per hospitalization | $9,709 | Gamma (alpha = 25; beta = 388) |
| Cost per ambulance hour | $174 | Gamma (alpha = 25; beta = 7) |
| Utility values if no event | 0.37 | Beta (alpha = 37; beta = 63) |
| Decrease in utility from emergency department visit | 0.018 | Beta (alpha = 1.8; beta = 98.2) |
| Decrease in utility from hospitalization | 0.06 | Beta (alpha = 6; beta = 94) |

Notations: SE: standard error

- 1. Sensitivity analyses

While parameter uncertainty is considered by the probabilistic analyses, several sensitivity analyses were conducted to examine the impact of changing one assumption/variable on the results (e.g., emergency department transport rate and costs for hospitalizations or emergency department visits are 20% more expensive than base case values). The table below presents the different sensitivity analyses which were conducted and their rationale.

Similar to the base case analysis, all sensitivity analysis were probabilistic, in which the model results were simulated 5,000 times with values from model input variables being drawn from distributions specific to each model parameter.

| **Sensitivity Analysis** | **Rationale and justification** |
| --- | --- |
|  |  |
| Urban based transportation and ambulance time data | There can be differences in the delivery of health care in rural settings compared to urban settings. These two sensitivity analyses provide model results specific to individuals treated in rural or rural settings based on the data collected as part of the Program. |
| Rural based transportation and ambulance time data |  |
| Assume 30% of people will be treated at home under the Program instead of being transported to the emergency department | The proportion of people treated at home under the Program instead of being transported to the emergency department was 60% based on 5,416 calls from the 5 Paramedic Partners. However, the proportion of people treated at home under the Program varies from 30% to 70%. This series of sensitivity analyses explore the impact of assuming different values of emergency department transport rate for the program. |
| Assume 50% of people will be treated at home under the Program instead of being transported to the emergency department |  |
| Assume 60% of people will be treated at home under the Program instead of being transported to the emergency department |  |
| Assume 70% of people will be treated at home under the Program instead of being transported to the emergency department |  |
| Assume emergency department to hospital rate for program is 66% (compared to 83% in the base case) | Since the Paramedic Partners could not connect their databases to hospital databases, an assumption was made regarding the percentage of emergency department visits that resulted in a hospitalization, which were assumed to be 83% for the Program (compared to 66% for the status quo). These sensitivity analyses look at the model results if alternative assumptions of emergency department to hospital transfer rates for the program are used (66%,75%, 90% and 100%). |
| Assume emergency department to hospital rate for Program is 75% (compared to 83% in the base case) |  |
| Assume emergency department to hospital rate for Program is 90% (compared to 83% in the base case) |  |
| Assume emergency department to hospital rate for Program is 100% (compared to 83% in the base case) |  |
| Intervention cost per call is $270 | The Program cost per 9-1-1 call impacts the overall cost savings, return on investment and cost-effectiveness. The Program cost per 9-1-1 call considered in the base case analysis was based on average costs observed among the five Paramedic Partners. This sensitivity analysis explores the impact of changing the Program costs of $493 per 9-1-1 call by +/-20% and using the lowest ($270) and highest ($3,869) cost per 9-1-1 call observed amongst the Paramedic Partners. |
| Intervention cost per call 20% higher than in base case analysis |  |
| Intervention cost per call 20% lower than in base case analysis |  |
| Intervention cost per call is $3,869 |  |
| Amortizing training costs over three years | Paramedic training may have benefits beyond the years analyzed in this study. Therefore, it could be argued that costs should be amortized over the “useful life” of the training. In the absence of data, we are making the conservative assumption here that paramedic palliative training will have a useful life of 3 years. |
| Include 2019/2020 data for transport rate and ambulance time | Data for 2019/2020 was only in four jurisdictions. Therefore, it was excluded from the base case analysis. This sensitivity analysis explores the impact of including this data. |
| Assume 95% transport to the emergency department for the status quo in the absence of the Program | The base case analysis assumes that the emergency department transport for the status quo rate is 0.90 based on data from New Brunswick (NB) before the implementation of the Program in NB. While three Paramedic Partners validated this assumption, one Paramedic Partner mentioned a higher (95%) emergency department transportation rate for the status quo while another Paramedic Partner indicated a lower (87%) emergency department transportation for the status quo. These sensitivity analyses look at the impact of alternate assumptions of the emergency department transportation rates (87%; 95%) for the status quo compared to the base case analysis using 90%. |
| Assume 87% transport to the emergency department for the for the status quo in the absence of the Program |  |
| Unit costs 20% higher than in base case analysis | Unit costs used in the base case analyses for palliative care hospitalization, and emergency department visits are based on Canadian averages. However, there are variations in the costs of hospitalization and emergency department visits between Canadian provinces. This sensitivity analysis looks at the impact of changing the unit costs +- 20%. |
| Unit costs 20% lower than in base case analysis |  |

1. Cost survey template

A cost survey was completed by each Paramedic Partner to calculate the cost of implementing and running the Program. The survey which is presented thereafter was divided into two main components (e.g., Program costs and Ambulance costs) as described below.

1. The Program costs were derived based on the information below.
2. Training Program (sections 1.1 and 1.2): the survey asked questions about the type of training used (Learning Essential Approaches to Palliative Care [LEAP] or other (section 1.1 in the survey below). The survey also asked whether the training was incorporated into regular educational activities (section 1.2).
3. Program project costs (section 1.3): each program was asked to provide their annual project costs broken into the following categories: compensation (e.g., salaries related to project leader, manager); meeting and travel (e.g., travel for meetings or for providing the training); professional/education services (e.g., information technology services, paramedic training); equipment (e.g., software); and other costs
4. Other costs (section 1.4): this section primarily focussed on collecting information on the costs related to backfill of positions and compensation given to paramedics for their training time if applicable (e.g., paramedic training occurred during work or off-hours)
5. Retraining costs (section 1.5): this section collected information to the cost of retraining in the Program cost estimates.
6. Training volume (section 2.1-2.3): The survey included questions around training volume for each Paramedic Partner which combined with information from section 1.4 was used to determine a cost per paramedic trained.
7. The ambulance costs were based on the following information.
8. Total annual amortized cost of ambulances in their jurisdiction (section 3.1)
9. Annual personnel costs (e.g., paramedic salaries; section 3.2)
10. Annual operating costs of ambulances (e.g., fuel, maintenance costs; section 3.3).
11. Total number of hours that ambulances were used (section 3.4)
12. Number of hours that ambulances were available (section 3.5) during the last fiscal year. This data was asked in order to derive the hourly cost of ambulance use.


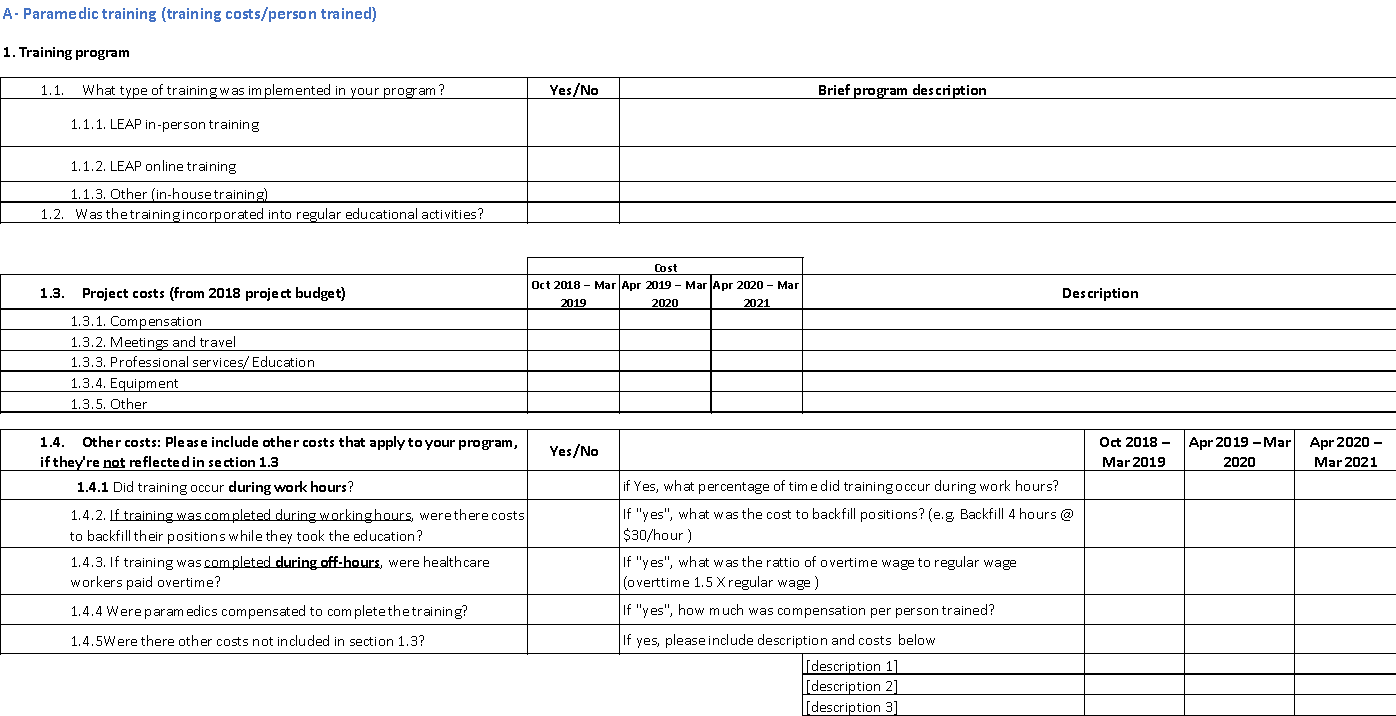


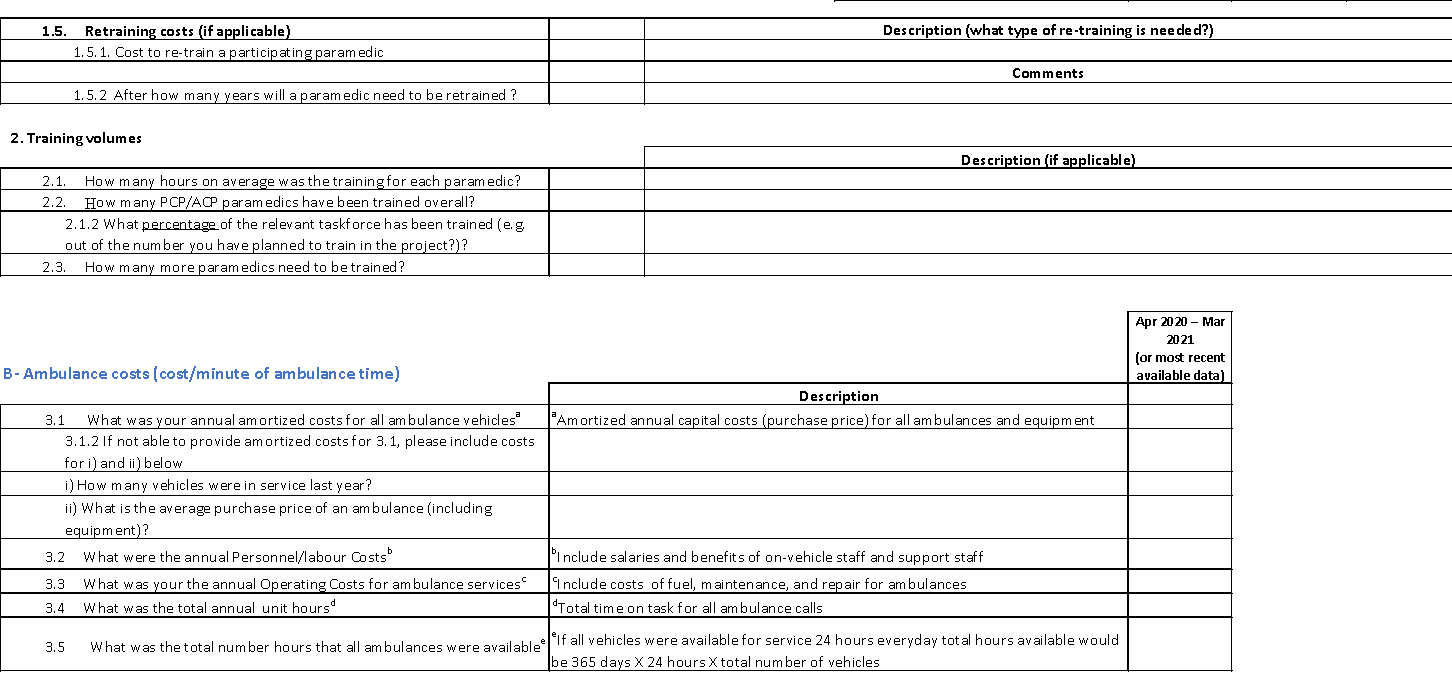


**REFERENCES**

1. Canadian Agency for Drugs and Health Technologies (CADTH). Guidelines for the Economic Evaluation of Health Technologies: Canada — 4th Edition [Internet]. 2017. Available from: <https://www.cadth.ca/guidelines-economic-evaluation-health-technologies-canada-0>.

2. Drummond M, Sculpher MJ, Claxton K, Stoddart G, Torrence G. Methods for the Economic Evaluation of Health Care programmes: Oxford University Press; 2015.

3. Cameron C, Carter A, Crick S, Edlund D, Pooler C, Arab M, Anderson E. Paramedics and Palliative Care: Bringing Vital Services to Canadians Change Package [Internet]. 2022 [cited Februrary 24 2023]. Available from: <https://www.healthcareexcellence.ca/media/it1dax2l/paramedics-palliative-care-change-package-eng.pdf>.

4. Canadian Institute for Health Information. Access to palliative care in Canada: 2023. Ottawa [Internet]. 2023 [cited August 28 2023]. Available from: <https://www.cihi.ca/sites/default/files/document/access-to-palliative-care-in-canada-2023-report-en.pdf>].

5. Canadian Broadcasting News. Paramedics hope to help palliative patients stay at home longer [Internet]. 2020 [cited February 24, 2023]. Available from: <https://www.cbc.ca/news/canada/new-brunswick/ambulance-new-brunswick-paramedics-palliative-susan-dugas-1.5420377>.

6. Funk LM, Mackenzie CS, Cherba M, Del Rosario N, Krawczyk M, Rounce A, et al. Where would Canadians prefer to die? Variation by situational severity, support for family obligations, and age in a national study. BMC Palliat Care. 2022;21(1):139.

7. Dzingina MD, McCrone P, Higginson IJ. Does the EQ-5D capture the concerns measured by the Palliative care Outcome Scale? Mapping the Palliative care Outcome Scale onto the EQ-5D using statistical methods. Palliat Med. 2017;31(8):716-25.

8. Church J, Goodall S, Norman R, Haas M. The cost-effectiveness of falls prevention interventions for older community-dwelling Australians. Aust N Z J Public Health. 2012;36(3):241-8.

9. Ghatnekar O, Bondesson A, Persson U, Eriksson T. Health economic evaluation of the Lund Integrated Medicines Management Model (LIMM) in elderly patients admitted to hospital. BMJ Open. 2013;3(1).

10. Health Quality Ontario. Time spent In Emergency Departments [Internet]. 2023 [cited Augist 29 2023]. Available from: <https://www.hqontario.ca/system-performance/time-spent-in-emergency-departments>.

11. Canadian Institute for Health Information. Hospital spending: Focus on the emergency department. Ottawa Ontario,. [Internet]. 2020 [cited Feb 23 2023]. Available from: <https://www.cihi.ca/sites/default/files/document/hospital-spending-highlights-2020-en.pdf>.

12. Canadian Institute for Health Information. Patient Cost Estimator [Internet]. 2022 [cited Februrary 23 2023]. Available from: <https://www.cihi.ca/en/patient-cost-estimator>.

13. Table 18-10-0006-01 Consumer Price Index, monthly, seasonally adjusted [Internet]. 2022 [cited Februrary 23 2023]. Available from: <https://www150.statcan.gc.ca/t1/tbl1/en/tv.action?pid=1810000601>.

14. Briggs AS, M,Claxton,C. Decision Modelling for Health Economic Evaluation: Oxford University Press; 2006.

15. Binder L, Ghadban M, Sit C, Barnard K. Health Technology Assessment Process for Oncology Drugs: Impact of CADTH Changes on Public Payer Reimbursement Recommendations. Curr Oncol. 2022;29(3):1514-26.
